# Fidelity of the implementation of antirabies vaccination for dogs and cats in the Plurinational State of Bolivia

**DOI:** 10.1371/journal.pntd.0014535

**Published:** 2026-07-10

**Authors:** Andrés Rodrigo Gómez-Vidal, Héctor Vélez Santamaría, Nathalia M. Correa-Valencia, Carlos Rojas-Arbelaez

**Affiliations:** 1 Grupo Epidemiología, Facultad Nacional de Salud Pública, Universidad de Antioquia, Medellín, Colombia; 2 Grupo de Estudios en Pedagogía Infancia y Desarrollo Humano, Faculty of Education, Universidad de Antioquia, Medellín, Colombia; 3 CENTAURO Research Group, Faculty of Agricultural Sciences, Universidad de Antioquia, Medellín, Colombia; University of Massachusetts Amherst, UNITED STATES OF AMERICA

## Abstract

The rabies virus is a serious global public health threat that primarily affects vulnerable populations in rural areas. Despite vaccination efforts, some countries, such as the Plurinational State of Bolivia, continue to report human fatalities and confirmed outbreaks in dogs caused by this virus. This study evaluated the fidelity of the implementation of an antirabies vaccination campaign for dogs and cats in the southern area of the Cercado municipality, Cochabamba (Bolivia), in 2021 to identify implementation gaps that may undermine rabies control as a public health strategy in endemic regions. A cross-sectional observational study was conducted using a purposive sampling approach, combining quantitative and qualitative methods through a parallel nonconvergent design. Seventeen health centers in the southern area of the study municipality were examined via a 27-point checklist to assess the fidelity of the implementation of the vaccination campaign in accordance with current national regulations. Additionally, semistructured interviews were conducted with 46 intervention providers and 108 dog and cat owners to explore perceptions regarding institutional responsibility and campaign functioning, following COREQ guidelines. All health centers demonstrated low adherence to the intervention content, with compliance rates below 80% in all participating establishments. A total of 70.6% (12/17) of the centers achieved adequate coverage, 82.4% (14/17) maintained an appropriate vaccination frequency, and 64.7% (11/17) reported a sufficient duration. Factors related to the antirabies vaccination campaign (including values associated with the intervention, inner-setting conditions, expected efficacy, and communication and coordination strategies) were identified as both barriers to and facilitators of implementation fidelity in the study area. Dog and cat vaccination campaigns face infrastructure, socioeconomic, and institutional barriers, along with weak oversight and communication. Nonetheless, health officials and pet owners recognize rabies as a serious public health concern that requires urgent control measures.

## Author summary

Rabies is a fatal but preventable disease that disproportionately affects vulnerable populations in low-resource settings. Vaccinating dogs and cats is the most effective strategy for preventing human rabies, yet transmission persists in some endemic countries, including Bolivia. In this study, we evaluated how well a mass rabies vaccination campaign was implemented in a high-risk area of Cochabamba. We found that none of the 17 health centers fully adhered to national guidelines, revealing critical gaps in implementation. Major challenges included inadequate cold chain infrastructure, insufficient personnel, weak interinstitutional coordination, and limited communication with communities. Despite these barriers, both health workers and pet owners recognized rabies as a serious threat and expressed a willingness to support control efforts. Our findings highlight that the main barrier to eliminating rabies is not the lack of effective tools but the difficulty of delivering them consistently under real-world conditions. Strengthening coordination across the human and animal health sectors, improving logistics, and enhancing community engagement are essential for increasing vaccination coverage. Addressing these implementation gaps is critical to advancing rabies elimination and protecting both human and animal health.

## Introduction

Controlling zoonotic diseases poses significant public health challenges because of the complex interactions among humans, animals, and the environment [1]. Rabies virus (RABV) is a critical threat—100% fatal but preventable—and disproportionately affects vulnerable populations in marginalized rural areas [2]. Rabies remains a public health threat, particularly in low-resource settings, where social determinants such as poverty, education, and access to health services influence both exposure risk and the effectiveness of preventive interventions [3]. It causes an estimated 59,000 human deaths annually (95% CI: 25,000–159,000), with more than 95% occurring in Asia and Africa [3,4]. This wide confidence interval reflects the substantial uncertainty inherent in estimating the rabies burden in settings with limited surveillance infrastructure, and the true toll may be considerably higher given that the disease disproportionately affects rural and marginalized populations with limited access to health care systems—precisely the populations least likely to be captured in routine reporting [3]. Rabies control is fundamentally a One Health challenge: it requires simultaneous action at the human–animal interface and engaging the veterinary, public health, and community sectors under a shared framework of coordinated prevention [1]. The One Health approach recognizes that the health of humans, animals, and ecosystems is inextricably linked and that diseases at this interface cannot be effectively controlled through vertical, single-sector interventions alone [5]. In the context of canine rabies elimination, One Health principles translate into the joint mobilization of health authorities, veterinary services, community organizations, and educational institutions—all of which must function in a coordinated manner to achieve and sustain the vaccination coverage levels required to interrupt transmission. Despite widespread endorsement of this integrated approach in global rabies elimination frameworks, the practical implementation of cross-sectoral coordination at the local level remains poorly documented, particularly in Latin American endemic settings.

Between 2013 and 2016, human rabies cases occurred in Bolivia, Brazil, Haiti, and Peru. Across the Americas as a whole, approximately 7–12 human deaths from canine rabies were reported annually during this period, with ten deaths recorded in 2016 across all affected countries in the region [2]. While this represents a dramatic reduction from the more than 250 deaths per year recorded in the early 1980s, the persistence of fatalities in the 2010s underscores that elimination remains incomplete in several endemic countries, including Bolivia. In the Americas, approximately 1 million people receive postexposure prophylaxis annually, and the vaccination of 100 million dogs has led to an ~ 98% decrease in the number of human cases since 1983 [5,6]. Despite these advances, Bolivia remains endemic for RABV in dogs and cats, with 69 and 51 confirmed canine cases in 2020 and 2021, respectively [6,7]. Cercado (Cochabamba), one of the country’s most densely populated areas, has the highest incidence of rabies, especially in its impoverished southern zone [8]. In 2021, 34 of the 69 confirmed canine rabies cases in Cochabamba occurred in Cercado, with cases disproportionately concentrated in the southern zone—the most socioeconomically disadvantaged area of the municipality, characterized by high population density, informal housing, migrant communities, and limited access to veterinary and public health services [8]. This geographic disparity mirrors patterns documented in other Latin American settings, where rabies incidence and vaccination coverage gaps consistently cluster in urban peripheries and low-income areas, reflecting the intersection of poverty, informal pet ownership practices, and structural barriers to health service delivery [3,9]. In contrast, the northern zone of Cercado, which is more affluent and better served by public infrastructure, has historically reported lower rabies incidence, suggesting that socioeconomic determinants mediate both exposure risk and the effectiveness of preventive campaigns—a pattern that this study sought to characterize through an implementation fidelity lens. To address this endemic burden, the Bolivian Ministry of Health and Sports mandates an annual mass antirabies vaccination campaign for dogs and cats, which is conducted each September. In Cercado, the campaign is organized through a network of 17 first-level health centers, each responsible for planning and executing the campaign within its designated catchment area over a single mass vaccination day, followed by additional postcampaign vaccination sessions for animals not reached during the main event. Despite this annual programming, coverage consistently remains below PAHO’s 80% target [5]. The Bolivian campaign relies primarily on fixed vaccination posts located at health centers and mobile door-to-door brigades that cover areas with limited access; vaccines are administered free of charge, and both dogs and cats are targeted [7]. These strategies mirror the approaches used across most of Latin America, where mass vaccination campaigns combining fixed posts with active house-to-house outreach have been the cornerstone of canine rabies control since the 1980s [6]. The southern zone of Cercado is characterized by linguistic and cultural diversity, with a significant proportion of the population speaking Quechua alongside Spanish; campaign materials and messaging are delivered in both languages to enhance community reach [6].

The history of rabies control in the Americas reflects a progressive shift from reactive postexposure management toward proactive mass vaccination strategies targeting the canine reservoir. Since the 1980s, PAHO-coordinated campaigns have achieved remarkable reductions in human rabies deaths across the region, with the number of cases declining from more than 250 per year in the early 1980s to fewer than 10 annually by the 2010s [5,6]. However, this progress has been uneven: while several countries in South America have eliminated human rabies transmitted by dogs, others—including Bolivia, Haiti, and parts of Peru and Brazil—continue to report fatalities, reflecting persistent gaps between national elimination commitments and local implementation capacity [2]. These persistent gaps have increasingly attracted the attention of implementation scientists, who argue that the primary obstacle to elimination in endemic settings is not the lack of effective vaccines, protocols, or political will but rather the failure to deliver existing interventions with sufficient fidelity, coverage, and consistency at the point of care [3,10]. The implementation of fidelity—the degree to which an intervention is delivered as intended—has been identified as a critical but understudied determinant of public health campaign outcomes [10]. Research in this area has demonstrated that even well-designed vaccination campaigns can fail to achieve herd immunity thresholds when implementation deviates from the protocol at the operational level, whether due to cold chain failure, incomplete brigade composition, insufficient community outreach, or fragmented interinstitutional coordination [9,11]. Despite this evidence, implementation fidelity assessments of canine rabies vaccination campaigns in Latin America remain scarce, limiting the ability of program planners to identify and address the specific mechanisms through which campaigns fall short in endemic settings.

Most implementation-fidelity assessments of mass dog vaccination campaigns conducted to date originate from sub-Saharan Africa or South and Southeast Asia, leaving a marked gap in evidence from the Americas, where distinct health-system structures, socioeconomic conditions, and ecological contexts may produce different implementation challenges. The present study addresses this gap by applying Carroll’s conceptual framework for implementation fidelity together with the Consolidated Framework for Implementation Research (CFIR) to a mass dog and cat rabies vaccination campaign in an Andean city.

Therefore, the objective of this study was to evaluate the fidelity of the implementation of an antirabies vaccination campaign for dogs and cats in the southern area of the Cercado municipality, Cochabamba (Plurinational State of Bolivia), in 2021 to identify implementation gaps that may undermine rabies control as a public health strategy in endemic regions.

## Materials and methods

### Ethical approval and consent to participate

This study was approved by the Research Ethics Committee of the Universidad de Antioquia (Act 298, Dec 9, 2022; ratified Dec 16) and the Cochabamba Departmental Program for Rabies and Leishmaniasis Control (Dec 8, 2022). The procedures followed national and international ethical guidelines. Written informed consent, based on the Declaration of Helsinki, the Nuremberg Code, and Colombian Law 100, was obtained, respecting nonmaleficence, beneficence, and justice.

### Study area and design

**Fig 1:**
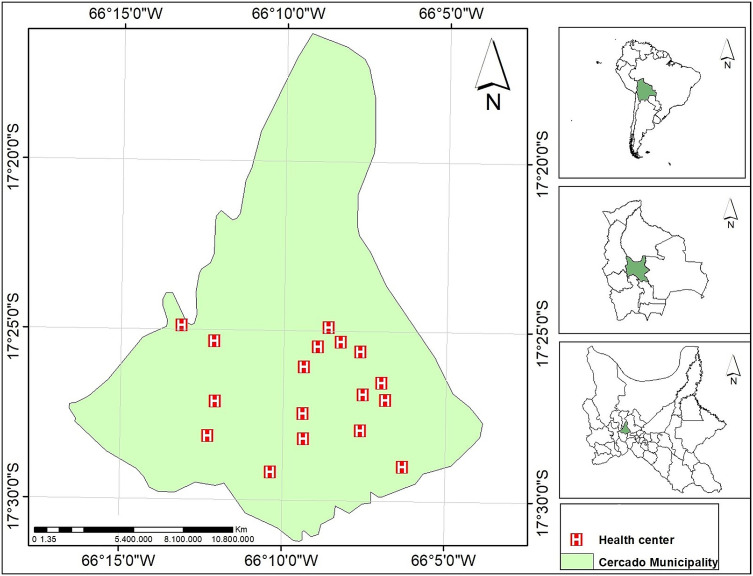
Location of the 17 health centers in the Cercado municipality (Cochabamba Province, Plurinational State of Bolivia), 2021. Base map shapefile obtained from Natural Earth (public domain): https://www.naturalearthdata.com/downloads/. According to Natural Earth terms of use (https://www.naturalearthdata.com/about/terms-of-use/), these data are in the public domain and are free to use, modify, and distribute without restriction and are therefore compatible with the CC BY 4.0 license.

The study was conducted in the municipality of Cercado, which is in Cochabamba Province within Bolivia. Cercado lies in the southwestern region of the province and is part of the metropolitan area along with the municipalities of Colcapirhua, Quillacollo, Tiquipaya, and Sacaba. It borders Chapare Province to the north and east, Quillacollo to the west, and Capinota and Esteban Arze provinces to the south (Fig 1). The municipality hosts 17 health centers that provide first-level care. These 17 first-level health centers are the operational units responsible for planning and executing the annual mass antirabies vaccination campaign, working with the decision-making entities (ETDs) that coordinate it; the levels of the Bolivian health system and the institutional structure within which the campaign is delivered are described under Description of the intervention.

A cross-sectional observational study was conducted using a purposive sampling approach, combining quantitative and qualitative methods through a parallel, nonconvergent design, after the antirabies vaccination campaign for dogs and cats was held in September 2021. The collection of information was carried out during the last three months of 2022. The retrospective nature of the data collection was deliberate and planned: given that the study aimed to evaluate a campaign that had already been executed, retrospective documentary review was the appropriate and only feasible method for capturing the evidence of what had actually been implemented. Health centers were notified in advance of the evaluation visits and were asked to make all the documentation generated during and after the September 2021 campaign available, including vaccination registries, meeting minutes, official correspondence, and WhatsApp group records with OTBs. A recognized challenge of retrospective documentary methods is incomplete or inconsistent record-keeping at the facility level, which may result in underreporting of activities that were carried out but not formally documented. To address this, the research team applied a conservative verification protocol: any activity for which documentary evidence was absent or insufficient was recorded as “NO” (not completed), regardless of verbal reports from personnel. This approach may have resulted in some underestimation of actual adherence but ensured that fidelity scores reflected verifiable implementation rather than self-reported compliance.

### Description of the intervention

The intervention under evaluation was the annual mass antirabies vaccination campaign for dogs and cats conducted in September 2021 in the southern area of the Cercado municipality, Cochabamba, Bolivia. This campaign is a routine public health intervention mandated by the National Prophylaxis Standard [7] and is operationalized through the Manual for Canine and Feline Rabies Vaccination Campaigns issued by the Ministry of Health and Sports [12]. It is designed to achieve and sustain a minimum vaccination coverage of 80% of the estimated dog and cat population—the threshold established by PAHO as necessary to interrupt canine-to-human transmission [5]. The 17 first-level health centers in the study area serve as the operational units responsible for planning, coordinating, and executing the campaign within their respective catchment areas. Each center is expected to (1) assemble complete vaccination brigades, including a vaccinator, channeler, and registry personnel; (2) maintain an adequate cold chain for vaccine storage and transport; (3) promote the campaign at least one month in advance through community communication channels; (4) conduct daily vaccination sessions of at least eight hours during the campaign period; and (5) ensure postcampaign vaccination availability for animals not reached during the mass event. Decision-making entities (ETDs)—including the Departmental Health Service (SEDES), the Cercado Health Network I, and the Municipal Zoonosis Center—are responsible for coordination, resource allocation, and supervision, whereas territorial base organizations (OTBs) play a key role in community mobilization and dissemination.

The campaign is delivered through the structure of the Bolivian public health system, which is organized into three levels of care: first-level centers (doctor’s office, consulting room, polyclinic, multispecialty clinic, and health center) deliver primary and preventive care, including immunization campaigns; second-level facilities (hospitals) provide specialized outpatient and inpatient services; and third-level hospitals concentrate highly complex care. The 17 first-level health centers in Cercado are therefore the sole facilities responsible for planning and executing the campaign at the community level, with no involvement of second- or third-level facilities. The veterinary sector operates through a separate institutional structure: the SEDES coordinates zoonosis programs, the Municipal Zoonosis Center oversees operational logistics, and the Cochabamba College of Veterinarians is the principal professional body for veterinary practitioners; however, veterinary professionals are not formally integrated into the campaign execution structure, which is managed exclusively through the human health system.

### Analytical and reporting frameworks

Two complementary frameworks guided the analysis and reporting of the findings. First, Carroll’s conceptual framework for implementation fidelity [10] was selected to structure the quantitative component. This framework defines implementation fidelity as the degree to which an intervention is delivered as intended and operationalizes it through four constructs: adherence to intervention content, coverage of the target population, frequency of delivery, and duration of the intervention. Carroll’s framework was chosen because it provides a structured, reproducible method for measuring the extent of implementation against a predefined protocol—in this case, the national vaccination standard—and has been validated across diverse public health intervention contexts. Second, the CFIR [13] guided the qualitative component. CFIR offers a comprehensive taxonomy of multilevel constructs—spanning the intervention, inner setting, outer setting, individuals involved, and implementation process—that explain why implementation succeeds or fails in a given context. Whereas Carroll’s framework addressed the “what” of implementation (the extent to which the campaign was delivered as planned), the CFIR addressed the “why” (the organizational, contextual, and individual factors driving or hindering that delivery). Together, these frameworks enabled a parallel nonconvergent mixed methods design in which quantitative fidelity scores and qualitative barriers and facilitators were generated independently using their respective frameworks and subsequently integrated during interpretation, producing a comprehensive and theoretically grounded diagnosis of implementation performance. The qualitative component was reported following the Consolidated Criteria for Reporting Qualitative Research [COREQ; 14], a 32-item checklist for interviews and focus groups. In brief, the CFIR served as the analytical framework that structured the qualitative inquiry, whereas the COREQ functioned solely as a reporting standard for the transparent description of the qualitative methods and findings. The full COREQ checklist with item-by-item responses is available from the corresponding author upon request.

### Quantitative component

All 17 first-level health centers in southern Cercado were evaluated for implementation fidelity. Maps and aerial photographs were used to delimit the geographic catchment area of each health center and to verify the spatial distribution of vaccination points within each center’s jurisdiction, enabling the research team to cross-reference the reported population coverage against the actual territorial extent of each center’s operational area. These centers, responsible for planning and executing the rabies vaccination campaign for dogs and cats in September 2021, provided retrospective documentary data for analysis. The implementation of fidelity was assessed using Carroll’s conceptual framework [10], which operationalizes implementation fidelity through four measurable constructs: adherence to intervention content, coverage of the target population, frequency of delivery, and duration of the intervention. This framework was selected because it provides a structured, quantifiable assessment of the degree to which a public health intervention is delivered as intended, thereby enabling objective identification of implementation gaps. Carroll’s framework guided the construction of the 27-point checklist and the subsequent scoring of each health center against the national vaccination protocol, producing a quantitative measure of fidelity for each of the four constructs. Defining adherence as the extent to which programs follow planned specifications, a 27-point checklist, based on the National Prophylaxis Standard [7] and the Manual for Canine and Feline Rabies Vaccination Campaigns issued by the Ministry of Health and Sports [12], evaluated four components, namely, content (19 questions), coverage (2), frequency (4), and duration (1), accounting for 26 of the 27 items. The remaining item corresponded to a documentary verification check confirming that the required administrative records were available and complete at the time of the evaluation visit; this item was assessed transversally across all four components and did not map exclusively to any single construct (S1 Appendix, in Spanish). Each checklist was coded for confidentiality. The 17 centers were notified of the visit dates for data collection and verification. Compliance was assessed through documentation, meeting minutes, official letters, and messages in WhatsApp groups with social and territorial base organizations (OTBs, by their name in Spanish), with responses recorded as “YES” if the activity was completed and “NO” otherwise. The binary response format was applied strictly: each checklist item required complete fulfillment of the specified criterion to be recorded as “YES”; partial delivery—for example, a vaccination brigade present but missing one required member, or a cold chain maintained for part but not all of the campaign period—was recorded as “NO”. This approach was adopted to ensure internal consistency and comparability across centers and to align with the pass/fail nature of the national standard against which fidelity was being assessed. We acknowledge that this strict binary operationalization does not capture gradations of partial compliance and may result in some underestimation of overall implementation effort. This is a recognized limitation of binary fidelity assessment instruments, and future studies may benefit from including a graduated response scale (e.g., fully met, partially met, or not met) to capture nuanced differences in delivery quality across checklist items. Binary YES/NO scoring was a deliberate methodological choice consistent with the protocol-based nature of the evaluation: each checklist item was operationalized as a discrete, verifiable activity specified in the national vaccination standard, for which documentary evidence either existed (YES) or did not (NO). Activities for which partial or incomplete evidence was found—for example, a vaccination log that covered only part of the campaign period or a cold chain record that was incomplete—were recorded as NO, as they did not meet the full specification required by the protocol. This conservative approach ensured comparability across centers and alignment with the binary compliance framework established by the Ministry of Health and Sports. We acknowledge that this scoring method does not capture degrees of partial implementation; however, given that the primary objective was to assess adherence to a defined protocol rather than to quantify the volume of activities delivered, the binary scale was the most appropriate and reproducible instrument for this purpose. The geographic distribution of the 17 health centers evaluated is shown in Fig 1, providing an essential context for interpreting the spatial scope of the intervention area. As the study assessed implementation fidelity across decentralized health centers, visualizing their location within the southern zone of the Cercado municipality is important for understanding how logistical barriers, variations in resource allocation, and differences in campaign performance may be influenced by geographical factors.

### Qualitative component and reporting guidelines

The qualitative component characterized the organizational barriers and facilitators underlying the fidelity gaps quantified above, using the CFIR constructs described in the preceding section. The qualitative data were collected through individual in-depth interviews (IDIs) conducted with 46 participants from key health and zoonosis entities (e.g., SEDES, Cercado Health Network I, local health centers) and 108 adult dog and cat owners residing in southern Cercado. All participants provided written informed consent prior to participation; identities and institutional affiliations were kept confidential throughout the study. The interviews were conducted by a team of four trained final-year veterinary students from the Faculty of Veterinary Sciences at Universidad Mayor de San Simón (UMSS), supervised by the principal investigator and supported operationally by the Municipal Zoonosis Center. Prior to data collection, all the interviewers underwent a two-day training workshop covering CFIR-guided qualitative inquiry, interview instruments, active listening and probing techniques, digital recording procedures, and ethical protocols for handling participant information. None of the interviewers had a prior professional or personal relationship with the participants, and no ETD personnel were involved in interviewing their own institutional colleagues to minimize social desirability bias. Researcher reflexivity was addressed through a structured debriefing protocol after each day of fieldwork: interviewers documented potential sources of bias in field notes, which were reviewed collectively with the principal investigator to identify and mitigate emerging analytical preconceptions. Interviews with ETD personnel were conducted at their respective health center facilities, in private consultation rooms, by prior appointment during working hours. Interviews with dog and cat owners were conducted at participants’ homes or at the location of their choice within the catchment area, following prior scheduling through community liaisons and OTB representatives. All the interviews were conducted in Spanish (or Spanish-Quechua as needed) and digitally recorded with participant consent; the mean interview duration was approximately 25–40 minutes for ETD personnel and 15–25 minutes for pet owners. Recordings were transcribed verbatim in Spanish; selected quotations were maintained in Spanish throughout the analysis to preserve contextual meaning and translated into English for reporting purposes only. Separate semistructured interview guides were developed for ETD personnel and for dog and cat owners (S2 and S3 Appendix), each grounded in the CFIR constructs relevant to each participant group. Pet owner locations were mapped using Google Earth to determine the catchment area distribution and proximity to health facilities. Data saturation was assessed iteratively: After each set of five interviews, two independent coders reviewed emergent themes and flagged the appearance of new codes. Saturation was considered reached when no new themes or subthemes emerged across three consecutive review cycles; this occurred at approximately the 38th ETD interview and the 90th pet owner interview. The remaining interviews were completed to ensure adequate representation across health centers and community zones. A coding framework aligned with the four CFIR construct domains was applied independently by two researchers; discrepancies were resolved through consensus discussion, and a third researcher served as an arbitrator for unresolved disagreements, ensuring analytical credibility. Member checking was conducted by returning a written summary of preliminary findings to a purposively selected subset of six ETD participants (representing different institutional levels: SEDES, Health Network, and local centers) and five pet owners. Participants were asked to review the accuracy, completeness, and interpretive fairness of the summary and provide written or verbal feedback within seven days; minor clarifications regarding institutional roles and campaign logistics were incorporated, and no substantive changes to core findings were needed.

Triangulation across participant groups, data sources (interviews and documentary review), and analytical frameworks (CFIR coding and quantitative fidelity scores) enhanced the trustworthiness of the findings. The full set of transcribed quotations in the original Spanish is available from the corresponding author upon request.

Checklist data were entered into Microsoft Excel 365, cleaned, and analyzed using descriptive statistics with absolute and relative frequencies. For each health center, compliance rates per construct (content, coverage, frequency, and duration) were calculated according to Carroll’s framework, with a threshold of ≥80% used to define adequate adherence, in line with the PAHO targets for rabies vaccination coverage.

For the qualitative component, the interview transcripts were analyzed using a framework analysis approach structured around the CFIR construct taxonomy, following five analytical stages: familiarization, coding, building an analytical framework, charting, and interpretation [15]. In the familiarization stage, all the transcripts were read in their entirety in the original Spanish by both researchers independently before any coding was applied. In the coding stage, each researcher generated an initial set of open codes by annotating recurring ideas, phrases, and expressed experiences directly on the transcripts; the codes were both deductive (grounded in the four CFIR construct domains established a priori) and inductive (emerging from the data). The two independent coding sets were compared, and discrepancies were resolved through consensus discussion; a third researcher served as an arbitrator for unresolved disagreements, ensuring analytical credibility. In the framework-building stage, finalized codes were mapped onto the four CFIR construct domains: (1) values associated with the intervention, (2) inner setting (organizational context, resources, and coordination), (3) expected efficacy of the intervention, and (4) communication and coordination strategies. Codes that recurred across participants but did not fit the *a priori* domains were retained as emergent subcategories and reported within the most conceptually proximate construct. In the charting stage, an analytical matrix was constructed in which rows represented individual participants and columns represented CFIR construct domains; each cell was populated with summarized evidence (quotations and paraphrases), enabling systematic cross-participant and cross-group comparisons. In the interpretation stage, patterns of barriers and facilitators were identified within and across participant groups by comparing cell entries across the matrix; dominant themes, exceptions, and contradictions were noted and resolved through reexamination of the original transcripts. Finally, the qualitative findings were triangulated with the quantitative fidelity scores by mapping identified barriers and facilitators onto the specific Carroll constructs where low adherence had been documented. This integration step — in which contextual barriers and facilitators identified via the CFIR were mapped onto the fidelity gaps quantified by Carroll’s framework — constituted the interpretive synthesis of the parallel nonconvergent mixed-methods design, allowing a richer understanding of not only where implementation fell short but also why.

## Results

### Fidelity estimation

**Fig 2:**
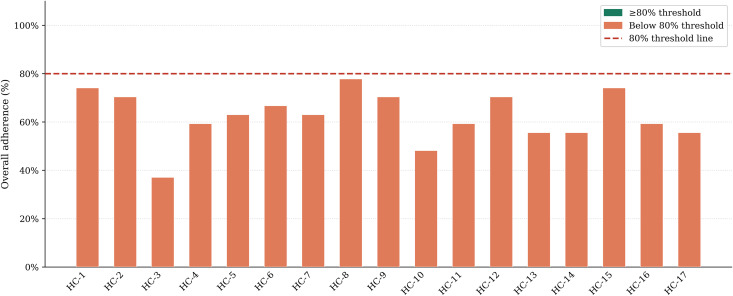
Adherence to vaccination campaign planning and execution in 17 health centers located in the Cercado municipality (Cochabamba Province, Plurinational State of Bolivia), 2021. HC = health center.

All 17 health centers were evaluated via a 27-point checklist covering content, coverage, frequency, and duration—the four constructs of Carroll’s conceptual framework for implementation fidelity. None achieved overall adherence above 80% in the planning and execution of the 2021 rabies vaccination campaign. The adherence levels across centers in southern Cercado are shown in Fig 2. The results below are organized by Carroll’s four fidelity constructs.

#### Content.

**Fig 3:**
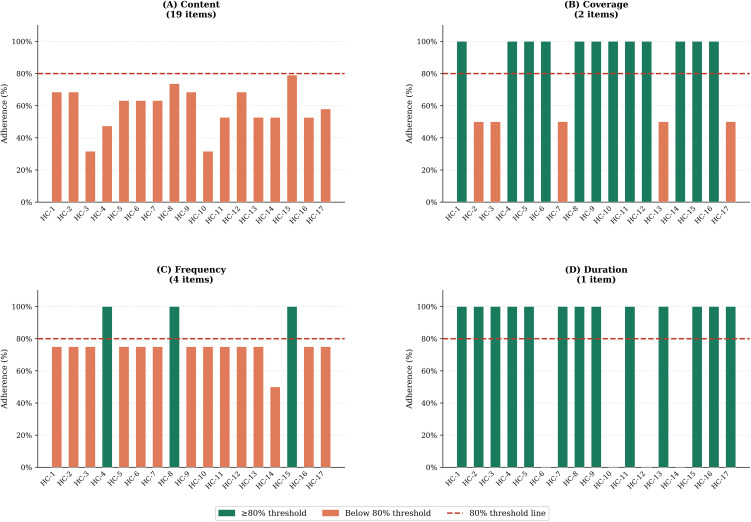
Adherence to the content (A), coverage (B), frequency (C), and duration (D) constructs during vaccination campaign planning and execution in 17 health centers located in the Cercado municipality (Cochabamba Province, Plurinational State of Bolivia), 2021. HC = health center.

All 17 health centers showed low adherence to the content of the vaccination campaign, with individual rates ranging from 32% (HC-3) to 79% (HC-15), and none reached the 80% threshold (Fig 3A). Most centers (13/17; 76.5%) lacked proper refrigerators, compromising the integrity of the vaccine cold chain. Incomplete vaccination brigades were reported by 88.2% (15/17) of the centers, primarily because of the absence of channelers. Loudspeakers for advanced campaign promotion were insufficient across most centers, and 47.1% (8/17) reported damaged coolers, further hindering the logistical execution of the campaign.

#### Coverage.

Twelve of the 17 health centers (70.6%) achieved full adherence (100%) to both coverage components—maintaining adequate vaccine availability during the mass campaign and ensuring continuity of vaccination during the postcampaign period (Fig 3B). The remaining five centers (29.4%: HC-2, HC-3, HC-7, HC-13, and HC-17) achieved only 50% adherence, meeting the vaccine availability requirement during the mass campaign day but failing to ensure postcampaign vaccination continuity for animals not reached during the main event.

#### Frequency.

Three of the 17 health centers (18%: HC-4, HC-8, and HC-15) achieved full adherence (100%) to all four frequency components (Fig 3C). Thirteen centers (76.5%) adhered to 75% of the frequency components—close to but below the 80% threshold—and one center (HC-14) achieved only 50% adherence. At the subcomponent level, advance campaign promotion launched at least one month prior was the most frequently unmet requirement, fulfilled by only 23.5% (4/17) of the centers. In contrast, the daily vaccination frequency and execution of a postcampaign vaccination day were the most consistently met components, which were followed by 94.1% (16/17) of the centers.

#### Duration.

A total of 76.5% (13/17) of health centers met the minimum 8-hour daily vaccination session requirement (Fig 3D). The four centers that did not meet this requirement (23.5%: HC-6, HC-10, HC-12, and HC-14) conducted shorter sessions, limiting the number of animals that could be vaccinated within each campaign day.

The qualitative analysis, guided by the CFIR and reported following COREQ guidelines (see Methods), identified barriers and facilitators for the planning and execution of the rabies vaccination campaign in southern Cercado. The data were triangulated across two main sources: interviews with 46 ETD personnel (S1 Table) and interviews with 108 dog and cat owners (S2 Table). This dual perspective allowed for a comprehensive understanding of the organizational and community factors influencing implementation fidelity. The findings presented below are organized according to the four CFIR construct domains that structured the qualitative analysis: values associated with the intervention, inner setting (organizational context, resources, and coordination), expected efficacy of the intervention, and communication and coordination strategies. Each theme emerged consistently across both participant groups, reinforcing its credibility. Triangulation confirmed a persistent disconnect between institutional planning capacity and community-level awareness and access. Data saturation was reached prior to the conclusion of data collection, indicating that further interviews were unlikely to yield new thematic insights.

### Identification of barriers and facilitators (CFIR-guided qualitative analysis)

**Fig 4:**
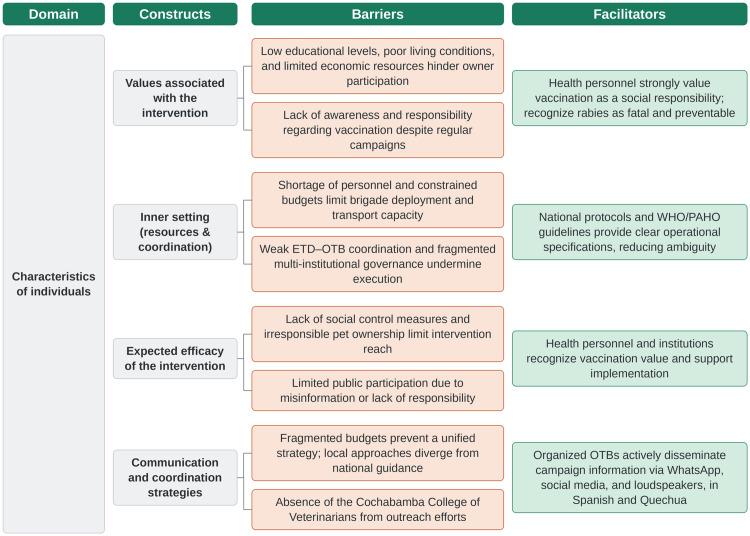
Barriers and facilitators are defined according to the Consolidated Framework for Implementation Research [13] in the southern area of the Cercado municipality, Cochabamba Province (Plurinational State of Bolivia), 2021. ETD (by its name in Spanish) = decision-making entity; OTB (by its name in Spanish) = territorial base organization.

Guided by the CFIR, barriers and facilitators were identified across four construct domains: values associated with the intervention, inner setting (organizational context, resources, and coordination), expected impact of the intervention, and communication and coordination strategies. These were explored during the planning and execution of the rabies vaccination campaign (Fig 4). Table 1 provides a structured summary of the barriers and facilitators identified per the CFIR construct domain as perceived by ETD personnel and dog and cat owners. The following section presents the supporting narrative, organized by the CFIR construct, with illustrative quotations from both participant groups.

**Table 1:** Summary of barriers and facilitators by the CFIR construct domain, as perceived by decision-making entity (ETD) personnel and dog and cat owners, southern Cercado municipality, Cochabamba (Bolivia), 2021.

CFIR Construct Domain	Source (% of testimonies)	Facilitators	Barriers
***Values associated with the intervention*** (beliefs about the worth and appropriateness of the intervention)	ETD personnel (34%)	Health personnel strongly value vaccination as a social responsibility and key public health measure; recognize rabies as fatal and preventable.	Low educational levels, poor living conditions, and limited economic resources among pet owners; migrant families leave pets unsupervised; general lack of awareness and responsibility regarding vaccination.
	Dog/cat owners	Recognition of rabies as a dangerous, fatal disease; motivation to protect animal and family health; willingness to inform neighbors and promote vaccination.	Persistent misinformation; limited knowledge of vaccination schedules and postbite procedures.
***Inner setting*** (organizational context: available resources, structural characteristics, and interinstitutional coordination)	ETD personnel (25%)	Existence of national protocols and global guidelines providing clear step-by-step specifications for campaign execution, reducing uncertainty at each implementation stage.	Shortage of personnel and constrained budgets limit brigade deployment capacity; weak ETD–OTB coordination and fragmented multi-institutional governance undermine resource allocation; lack of transport restricts geographic campaign reach.
	Dog/cat owners	Shared sense of responsibility across government levels and the community; willingness to collaborate in vaccination.	Limited awareness of campaign logistics and location of vaccination points; practical household barriers (securing animals, being present, poor perimeter security) reduce accessibility, particularly where children are primary pet caregivers.
***Expected impact of the intervention*** (perceived efficacy and reach of vaccination)	ETD personnel (20%)	Strong motivation among health personnel; empirical evidence from field observations confirms vaccinated animals do not develop rabies; vaccination seen as most effective prevention measure.	Lack of social control measures; irresponsible pet ownership (free-roaming animals); inconsistent coverage across municipal zones; poor OTB coordination limiting intervention reach.
	Dog/cat owners	Vaccine perceived as effective when properly administered; vaccination seen as a social contribution to disease control.	Limited public participation; knowledge gaps about vaccination schedules and effective disease prevention behaviors.
***Communication and coordination strategies*** (dissemination and interinstitutional coordination)	ETD personnel (21%)	Organized OTBs actively disseminate campaign information via WhatsApp groups, social media, and loudspeakers; messaging available in both Spanish and Quechua.	Fragmented budgets prevent unified strategy; local approaches diverge from national guidelines; low public awareness of vaccination timing and postbite procedures; notable absence of the Cochabamba College of Veterinarians.
	Dog/cat owners	Population perceives intervention information as generally accessible; community members willing to spread awareness informally among neighbors.	Need for increased campaign frequency and stronger media coverage; many community members still miss campaigns due to insufficient outreach.

ETD: decision-making entity; OTB: territorial base organization.

### Perception of decision-making entities

Four main themes were identified in the interviews conducted with the participants from the ETDs as follows.

#### Values associated with the intervention (34%).

ETD participants described vaccination as a socially responsible public health measure and recognized rabies as a fatal but preventable disease. Barriers in this domain were rooted in the socioeconomic conditions of the southern zone, including low educational levels, precarious housing, migrant mobility, and limited economic resources among pet owners, which collectively undermined community awareness and participation.

#### Inner setting—organizational context and resources (25%).

ETD participants identified structural organizational factors as the principal barrier to campaign execution, including personnel shortages, constrained budgets, fragmented ETD–OTB coordination, and lack of transport. The single facilitator in this domain was the existence of national protocols and WHO/PAHO guidelines, which reduced operational ambiguity by specifying the required steps for campaign delivery.

#### Expected efficacy of the intervention (20%).

ETD participants expressed strong collective belief in vaccination as the most effective measure to prevent rabies in both animals and humans, supported by direct field observations of vaccinated animals remaining disease-free. Barriers in this domain included limited public participation, irresponsible pet ownership (including free-roaming animals), and poor OTB coordination, which restricted the reach and impact of the campaign.

#### Communication and coordination strategies (21%).

Participants identified both facilitators and barriers in this domain. Organized OTBs that actively disseminated campaign information via WhatsApp groups, social media, and loudspeakers—in both Spanish and Quechua—were recognized as key facilitators of community reach. However, fragmented budgets across governmental levels prevented a unified strategy, local approaches from frequently diverging from national guidelines, and the notable absence of the Cochabamba College of Veterinarians from outreach efforts was identified as a significant gap.

### Facilitators related to the values associated with the intervention

Health personnel from various ETDs clearly understand the importance of vaccination, recognizing it as a key measure to prevent disease transmission and a vital act of social responsibility. Their efforts are seen as essential to public health, reinforcing the value of their role in protecting the community from rabies.

*“The first thing I think of is rabies is death. At that point, the messages we are trying to communicate, along with their importance, are that this is a deadly disease, one without a cure, but it is a disease that can be prevented” (ETD-1).*

### Barriers related to the values associated with the intervention

The participants noted that low educational levels, poor living conditions, and limited economic resources among dog and cat owners hinder campaign effectiveness. Many families in southern Cochabamba are migrants engaged in commerce, often leaving pets under the care of children in unsecured homes. A general lack of awareness and responsibility regarding rabies vaccination was also highlighted. Despite regular campaigns, participation remains low because of limited understanding and engagement among the population.

*“The difference between the southern and northern zones lies on one hand, in the sociocultural context, and on the other, in the political influences that have recently had a significant impact on the southern zone. From an economic standpoint, the more remote areas of the southern zone have experienced a higher incidence of rabies” (ETD-10).*

### Facilitators related to the inner setting (organizational context and resources)

ETD participants consistently cited the national vaccination protocol (Norma Nacional de Profiláxis Antirrábica) and the Ministry of Health’s campaign manual as key facilitators. These documents provided step-by-step operational guidance that reduced uncertainty and standardized brigade activities across the 17 health centers, with their alignment with the WHO and PAHO guidelines reinforcing their legitimacy as implementation references.

*“Where we work, our focus is more on the technical aspects, not so much the operational side. Our main goal is to ensure that the regulatory standards are properly followed, with particular emphasis on the correct administration of the vaccine” (ETD-2).*

### Barriers related to the inner setting (organizational context and resources)

While national protocols were recognized as facilitators, ETD participants described several structural factors that increased the complexity of campaign execution: personnel shortages that forced vaccinators to assume multiple roles simultaneously, budget constraints that limited brigade deployment, weak ETD–OTB coordination that fragmented planning, and the absence of formal transport arrangements that restricted geographic reach within catchment areas.

*“It is a bit complicated because people do not give it the necessary importance. There are very few leaders who truly show interest in vaccination efforts. From what I observed in the centers where I conducted training and awareness campaigns, only a handful of leaders genuinely cared about the issue of rabies vaccination” (ETD-6).*

*“There is even an economic limitation in the budget, including for refreshments” (ETD-43).*

### Facilitators related to the impact of the intervention

Most health system participants acknowledged that while they may not be the ideal personnel for implementation, they are strongly motivated by the belief that rabies vaccination is the most effective way to prevent disease in both animals and humans. This shared sense of purpose facilitates successful campaign delivery. They also agreed that vaccinated animals do not develop the disease, reinforcing vaccination as a safe and essential preventive measure to protect at-risk populations.

*“We would need more support from the zoonosis department because, in the end, we are healthcare personnel, not veterinarians. For example, during educational talks, people approach us with questions that we sometimes cannot fully answer” (ETD-31).*

*“We have observed in our current reality that vaccinated animals, for example, have not shown signs of rabies. Rabies cases have occurred only in animals that were not vaccinated” (ETD-1).*

### Barriers related to the impact of the intervention

Many participants noted that the lack of social control measures and irresponsible pet ownership hinder the reach of the intervention. They believe that enforcement measures should target those who fail to vaccinate their dogs or cats against rabies, particularly owners who allow their pets to roam freely, posing a public health risk. Additionally, poor organization and coordination of OTBs further exacerbate the lack of control, limiting the overall reach and effectiveness of the intervention.

*“Yes, I think it is important to regulate responsible pet ownership because there are very irresponsible owners. Even when vaccination is performed door-to-door, many owners are not willing to vaccinate their pets. It is like, “I’m not going to catch the animal; you do it,” for example” (ETD-14).*

### Facilitators of communication strategies

Owing to their organized structure, the participants clearly enhanced the reach of the intervention by actively participating in the dissemination and communication of the vaccination campaign at the zonal level. This is achieved through various communication channels, including social media and loudspeakers in community centers. Participants noted that campaign messaging is delivered in both Spanish and Quechua, which they identified as a facilitator of community reach in the linguistically diverse southern zone.

Notably, participants identified the use of WhatsApp groups linking ETD personnel with OTB representatives as a low-cost, effective coordination mechanism that facilitated real-time information sharing and campaign planning at the community level. This digital coordination channel was described as supplementing formal institutional communication structures, enabling faster dissemination of campaign schedules and logistical updates directly to community leaders.

*“I would like to mention that we have a WhatsApp group with representatives from each OTB, where we share updates on all the activities we have and plan accordingly. We also have “Mi Salud” doctors who help us with this issue. The OTB representatives are part of the WhatsApp group as well” (ETD-21).*

### Barriers to communication strategies

Communication efforts by ETDs are limited by fragmented budgets, preventing a unified strategy. While the national program provides a strong framework, local governments implement disconnected approaches that often diverge from national guidelines. As a result, publicity campaigns have a limited impact: many people remain unaware of what to do after an animal bite, the importance of rabies vaccination, or the timing and location of interventions. ETDs also highlighted the notable absence of the Cochabamba College of Veterinarians in supporting communication and outreach efforts, despite its key role in zoonotic disease control.

*“They are not enough, because if we focus only on the healthcare team providing educational talks, both at the institutional and community levels, we will not succeed without the support of the community” (ETD-18).*

*“They are not enough, because if we focus solely on the healthcare team giving educational talks, both at the institutional and community levels, we will not be effective without the community’s support” (ETD-18).*

*“The veterinary association is the one that should be working alongside us, but they are noticeably absent.” (ETD-1).*

### Perception of dog and cat owners

Four main themes were identified in interviews conducted with dog and cat owners based on documentation of 1,060 testimonies, as follows.

*Awareness of the disease* of rabies is a serious problem that requires immediate attention.

#### Interest and social trust in the intervention.

The intervention is carried out smoothly, and there is public interest in voluntarily collaborating. The motivation for vaccinating pets is focused primarily on animal welfare.

#### Responsibility for action.

Vaccination is a shared responsibility between different levels of government and the public.

#### Vaccination as a preventive measure.

Vaccination is the most effective and safest preventive measure to protect susceptible animals from rabies and to safeguard those living with them.

#### Relevance of communication strategy.

The communication strategies should be strengthened to better publicize the intervention.

### Facilitators of interest and social trust in the intervention

Animal welfare and the protection of loved ones are the primary motivations for the population when their dogs and cats are vaccinated against rabies. Additionally, there is interest in indirectly supporting the intervention through personal and social communication channels, allowing neighbors in different areas to ensure that their pets receive the rabies vaccination.

*“By informing my neighbors to vaccinate their pets during campaigns, taking my own pets for vaccination, and if I see an animal on the street, I report it so they can take it in. A lot also depends on how far they are able to reach” (pet owner-26).*

### Facilitators of vaccination as preventive measures

The population views the rabies vaccine as an effective tool for disease prevention. Therefore, vaccinating dogs and cats is considered an important preventive measure, as well as a social contribution to controlling the spread of the disease.

*“Indeed, vaccination is highly effective as a preventive measure, but only if it is properly managed and administered” (pet owner-33).*

### Facilitators of disease knowledge

The population recognizes rabies as a dangerous and fatal disease for both humans and animals. As a result, immediate action is considered crucial in the event of a dog or cat bite, including preventive measures such as washing the wound with soap and water and promptly seeking medical attention at a healthcare facility.

*“The primary measures I have been taught are to wash the wound thoroughly with plenty of water and soap, then go to the nearest health center, and if possible, capture the animal to check if it has been properly vaccinated” (pet owner 77).*

### Facilitators of responsibility for action

According to the population, the planning and execution of the rabies vaccination campaign in various zones and neighborhoods should be carried out not only by different levels of government but also by the collaboration of the public. Importantly, the participation of veterinarians as coresponsible for rabies immunization is not considered by the population, largely because of the absence of this professional group in the execution of the rabies vaccination campaign.

*“I suppose it starts with the community, beginning with the local government, then*

*our submunicipalities, and the OTB, right?” (pet owner-101).*

### Facilitators of the relevance of the communication strategy

The population perceives that information related to the intervention is accessible and readily available. As a result, little mention exists of the need to further strengthen communication strategies aimed at promoting intervention.

*“Maybe there should be more vaccination campaigns, not just twice a year but three times, with more publicity through the media to make it more widespread. There are people who are aware of the campaigns, but many still miss them” (pet owner-101).*

## Discussion

This study revealed critical implementation gaps in the fidelity of the rabies vaccination campaign in southern Cercado (Bolivia), highlighting systemic weaknesses that compromise rabies prevention as a public health strategy. Despite regulatory frameworks and a high level of awareness among health personnel, deficiencies in infrastructure, coordination, and communication—alongside limited engagement of key actors such as the veterinary sector—undermined campaign effectiveness. These findings underscore how logistical and institutional barriers can obstruct the delivery of essential preventive services, increasing the risk of human exposure in endemic areas. Critically, this study contributes to a growing body of implementation science evidence demonstrating that the gap between policy and practice—rather than the absence of effective vaccines or guidelines—is the primary obstacle to rabies elimination in endemic low- and middle-income settings [3,5]. By applying Carroll’s framework alongside the CFIR, this study moves beyond simply documenting whether coverage targets were met to systematically characterize the organizational, logistical, and sociocultural mechanisms that determine implementation quality—an approach that remains underutilized in the rabies control literature.

Rabies remains a major public health issue in Bolivia, especially in Cercado (Cochabamba), because of low vaccination coverage and high case numbers. In 2021 alone, five cases of human death were reported, highlighting ongoing challenges. In that same year, 69 cases of rabies in dogs were confirmed in Cochabamba, 34 of which occurred in Cercado.

Vaccination is the cornerstone of rabies prevention. However, the absence of a reliable census of the dog and cat population hinders effective planning and monitoring. Current estimation methods (e.g., one dog per four people) likely underestimate the true population, undermining coverage goals.

Quantitative results revealed suboptimal adherence, with fidelity ranging from 32 to 79%. Many centers lack key resources—cold chain equipment, full teams, and coolers—which are vital for vaccine preservation and delivery. This variability highlights unequal resources and inconsistent institutional support, calling for better standardization and coordination. These findings are consistent with evidence from comparable settings across Latin America and sub-Saharan Africa, where cold chain failures and incomplete vaccination teams have been repeatedly identified as proximal determinants of low campaign coverage [3,11]. The fact that no health center in the present study achieved adherence above 80%—the threshold established by the PAHO as necessary for herd immunity in dog populations—is particularly concerning given that Bolivia has been reporting confirmed canine rabies cases annually since before 2012 [7]. These findings reinforce the argument that coverage shortfalls in endemic settings are not random but rather predictable consequences of structural underresourcing of the primary health care level, which bears the operational burden of mass vaccination campaigns without commensurate logistical support. For program planners, these findings have direct operational implications. The cold chain failures documented here—damage to coolers in 47.1% of the centers and the absence of refrigerators in 76.5% of the centers—represent a systemic failure to allocate and maintain minimum infrastructure. Planners should consider context-adapted cold chain solutions that reduce the dependence on electricity-dependent refrigeration. A particularly relevant technological alternative is the use of thermotolerant inactivated rabies vaccines, which have been shown in clinical trials to retain sufficient immunogenicity when stored outside standard cold chain conditions [16]. Lankester and colleagues published multiple studies demonstrating the effectiveness of nonrefrigerated vaccine storage for canine rabies vaccination in resource-limited field settings, offering a concrete and evidence-based solution to one of the most prevalent infrastructure barriers identified in this study. In East Africa, passive cooling technologies such as locally manufactured clay pot coolers and phase-change material vaccine carriers have also successfully maintained vaccine temperatures in resource-constrained rural settings [3]. In the context of Bolivian, the adoption of thermotolerant vaccine formulations—combined with prepositioned insulated carriers distributed to each brigade before the campaign day—could substantially reduce cold chain dependency and improve content adherence without the need for capital investment in refrigeration infrastructure at every health center. Similarly, the brigade composition failures observed here—incomplete teams in 88.2% of centers—could be partially addressed through trained community health volunteer models, which have demonstrated effectiveness in Kenya [11] and could supplement understaffed health center teams during annual campaign peaks. The CFIR-guided qualitative component captured insights from ETD staff and community members on planning and execution. The interviews involved staff from the National Zoonosis Program, SEDES, the Municipal Zoonosis Center, and local health centers. Dog and cat owners were also included to capture community perceptions of rabies and the vaccination campaign.

The findings revealed that ETD personnel recognized rabies vaccination as a socially responsible public health measure. However, systemic issues—poor coordination, limited resources, and a lack of key stakeholder involvement—hindered implementation, and weak collaboration with OTBs further affected adherence to Ministry standards. These challenges mirror those reported in similar settings, where organizational and communication gaps undermine campaign success [9,11]. The multilevel governance fragmentation observed in this study—where national protocols exist but local implementation remains disconnected and underresourced—has been documented as a structural barrier to rabies control across diverse endemic contexts. In Kenya, Ferguson et al. [11] demonstrated that volunteer-based, community-embedded vaccination models can partially compensate for institutional coordination failures; however, sustainability remained contingent on sustained external support and clearly defined institutional roles. In Peru, Castillo-Neyra et al. [9] reported that even in outbreak contexts, households with dogs that were difficult to access, combined with owner reluctance, substantially reduced effective coverage—a pattern closely mirrored in the present study. Taken together, these findings suggest that the barriers to rabies vaccination fidelity are not idiosyncratic to Bolivia but rather reflect a broader pattern of implementation failure in settings where decentralized health systems lack the interinstitutional coordination mechanisms needed to translate national elimination targets into consistent local action [3,5]. For future campaign planning in Cercado and comparable settings, these findings suggest that improving inner-setting conditions—rather than modifying the intervention protocol itself—is the priority. Concretely, this means formalizing ETD–OTB coordination through written joint planning agreements, assigning clear accountability for each campaign stage to specific institutions, and establishing a shared monitoring mechanism that allows real-time tracking of brigade deployment and coverage across health centers. The WhatsApp-based coordination already in use by some ETDs in this study represents an existing, low-cost coordination infrastructure that could be systematized and extended to all 17 centers as a standardized communication protocol—converting an ad hoc facilitator into a structural feature of campaign planning.

Pet owners cited protecting both family and animal health as key motivators for vaccination; however, participation was often limited by knowledge gaps and logistical barriers with risk factors such as poor perimeter security and reliance on children for pet care. Despite the widespread recognition of rabies as a fatal disease, misinformation and low awareness persist, underscoring the need for culturally tailored education strategies. Involving veterinary professionals is key; evidence from Peru shows that their participation enhances public trust and may increase uptake [9]. The coexistence of high disease awareness and low vaccination participation observed among pet owners in this study is consistent with the risk perception–behavior gap documented in other neglected tropical disease contexts, where fatalistic attitudes, competing household priorities, and distrust of institutional health services mediate the translation of knowledge into protective action [3]. The reliance on children for pet handling identified here as a risk factor for household inaccessibility has not been previously reported in the rabies vaccination fidelity literature and may represent a locally specific barrier warranting targeted communication strategies. More broadly, these findings suggest that demand-side barriers—rooted in socioeconomic vulnerability, educational disadvantage, and geographic marginalization—interact with supply-side failures in infrastructure and coordination to produce the persistently low coverage levels observed in southern Cercado. Addressing only one dimension without the other is unlikely to result in sustainable improvements, a conclusion supported by the global evidence base on integrated approaches to rabies elimination [5,6]. In terms of practical implications for future operations, the persistent household accessibility barriers identified here suggest that door-to-door vaccination strategies—already partially implemented in the southern zone—should be complemented by appointment-based or evening and weekend vaccination options that accommodate working households and migrant families. Evidence from urban rabies control campaigns in other Latin American cities suggests that flexible scheduling and mobile vaccination units substantially improve coverage in dense, low-income neighborhoods [9]. Additionally, the finding that children are often the primary pet caregivers in migrant households points to the need for school-based rabies awareness programs that empower children to inform their families about campaign schedules and the importance of vaccination—a strategy that has been successfully piloted in other community-based disease prevention programs in the region.

The application of implementation research in this setting is novel and offers valuable insights into how public health interventions operate under real-world conditions. To our knowledge, this is the first study to apply Carroll’s conceptual framework for implementation fidelity specifically to a canine and feline rabies vaccination campaign in Latin America and one of very few to combine it with the CFIR to simultaneously measure the extent and explain the drivers of implementation gaps. Prior implementation research on rabies control has focused primarily on outcome metrics such as vaccination coverage and herd immunity thresholds [3,5] or on qualitative explorations of community-level demand barriers [9]. This study connects those approaches, producing a more complete implementation diagnosis that is directly actionable for program planners. Furthermore, the mixed-methods parallel nonconvergent design enabled the integration of institutional and community perspectives, revealing that the disconnect between health system capacity and community engagement is a shared, cross-cutting barrier—not simply a demand-side or supply-side problem in isolation. This methodological contribution has direct relevance for implementation research in other zoonotic disease control programs, particularly those operating through decentralized primary health care systems in endemic low-resource settings. Limitations of this study included the following: 1—The absence of national-level actors: The inability to interview Ministry of Health and Sports officials, national SEDES leadership, or national zoonosis program coordinators limits the depth of the inner-setting analysis. Decisions regarding budget allocation, personnel assignment, and vaccine supply chain management are made at the national level; their absence means that structural drivers rooted in national policy design or resource distribution frameworks cannot be fully characterized. Findings on organizational barriers should therefore be interpreted as reflecting local and departmental perspectives only—the full governance chain from policy to practice remains partially uncharted; 2—Nonvalidated data collection instruments: The 27-point fidelity checklist was developed specifically for this study and has not been formally validated in other settings. While grounded in the national vaccination standard, its psychometric properties (reliability and construct validity) have not been independently assessed. Fidelity scores should therefore be treated as indicators rather than as definitive measures, and cross-study comparisons using this instrument are not yet possible; and 3—Purposive sampling of pet owners: Selection on the basis of geographic proximity and availability rather than random probability sampling limits the statistical representativeness of qualitative findings for the broader pet-owning population of southern Cercado, and responses may overrepresent more accessible or more campaign-engaged households. However, as the qualitative component aimed to explore the range and nature of perceptions rather than estimate their prevalence, purposive sampling is methodologically appropriate; findings should be interpreted as illustrative of the types of barriers and facilitators present, not as population-level frequency estimates.

The findings of this study have direct relevance for the operationalization of One Health principles in rabies control. The One Health framework calls for joint, coordinated action across the human health, animal health, and environmental sectors, recognizing that problems at the human–animal interface cannot be effectively addressed through siloed, single-sector responses [1,5]. In this study, the absence of veterinary professionals from campaign planning and execution—explicitly identified by both ETD personnel and pet owners as a barrier—represents a fundamental failure of intersectoral collaboration. Veterinarians possess technical expertise in animal restraint, vaccine administration in animals, and zoonotic disease communication that health center personnel acknowledge they lack, particularly during community education sessions. Their systematic exclusion from the campaign reduces both technical quality and community trust in the intervention. Evidence from Peru [9] and Kenya [11] suggests that campaigns that formally integrate veterinary professionals and community animal health workers achieve higher coverage and stronger community uptake than those delivered exclusively through the human health system do. From a One Health perspective, the formal incorporation of the Cochabamba College of Veterinarians and veterinary training institutions into campaign planning—not as consultants but as coresponsible partners with defined roles and accountability—is not merely a logistical enhancement but a structural requirement for achieving the intersectoral collaboration that sustainable rabies elimination demands. Similarly, the weak engagement of OTBs, local governments, and community leaders documented in this study reflects a broader deficit in the horizontal, cross-sectoral governance mechanisms that One Health implementation requires. Strengthening these mechanisms—through formal interinstitutional agreements, joint planning tables, and shared monitoring systems—is essential to converting the One Health rhetoric endorsed by Bolivia’s national rabies program into operational reality at the municipal level.

These findings can guide stakeholders in strengthening rabies prevention. The identified limitations highlight areas for improving public awareness and control efforts. Key recommendations include reinforcing cold chain infrastructure, improving interagency coordination, standardizing communication, engaging with the Veterinary Medical Association, and promoting culturally tailored education. Additional priorities include setting annual vaccination targets, conducting pet censuses, and advancing implementation research to enhance outcomes and build public trust in rabies control. For the State, this supports more efficient investments in preventive and postexposure rabies control logistics. These recommendations are aligned with the WHO and PAHO strategic framework for the elimination of human rabies transmitted by dogs by 2030, which explicitly identifies implementation capacity strengthening—including surveillance system improvements, mass vaccination logistics, and community engagement—as priority action areas [5,6]. Despite having an established national prophylaxis standard and annual campaign infrastructure, Bolivia’s continued failure to reach the 80% vaccination coverage threshold exemplifies the implementation gap that this global target must close. The present study contributes empirical, locally grounded evidence on the specific mechanisms driving that gap in one of Bolivia’s highest-burden municipalities and offers a replicable methodological framework for other endemic countries seeking to diagnose and address implementation fidelity failures in their own rabies control programs.

## Conclusions and recommendations

This study identifies four actionable domains—values associated with the intervention, complexity, expected impact, and communication and coordination strategies—that implementers must address to improve the fidelity of rabies vaccination campaigns in endemic settings. First, health authorities should prioritize the reinforcement of cold chain infrastructure and ensure complete vaccination brigade composition before each campaign cycle, as deficiencies in these basic operational inputs were the primary drivers of low content adherence across all 17 health centers. Given that 76.5% of health centers lacked adequate refrigeration, the adoption of thermotolerant inactivated rabies vaccines—whose immunogenicity under nonrefrigerated storage conditions has been demonstrated in clinical trials—represents a high-priority and evidence-based recommendation to overcome this structural barrier without requiring capital investment in cold chain infrastructure at every health center. Second, interinstitutional coordination between decision-making entities and territorial base organizations (OTBs) must be formalized through joint planning protocols and shared accountability mechanisms; fragmented governance was consistently identified as a key barrier to campaign execution. Third, the Veterinary Medical Association and veterinary training institutions should be formally integrated into campaign planning and delivery, given that their absence was explicitly identified by both health personnel and community members as undermining public trust and technical quality. Fourth, communication strategies must be standardized across governmental levels, delivered in both Spanish and Quechua, and launched at least one month in advance of each campaign; the current fragmentation of messaging was found to directly limit community awareness and participation. Finally, implementers should establish routine pet censuses and set annual, facility-level vaccination targets to enable evidence-based planning and progress monitoring. Sustained investment in these areas is essential for closing the implementation gap and achieving lasting reductions in human and animal rabies in Cercado and comparable endemic municipalities.

## Supporting information

S1 Appendix Vaccination campaign fidelity checklist, a checklist used to assess campaign fidelity across 17 health centers, covering 27 items grouped into content, coverage, frequency, and duration. Language reflects local administrative and operational terms.
(PDF)

S2 Appendix Interview guide for decision-making entities (ETDs), a semistructured guide capturing the perceptions of health personnel and public officials. Wording preserves institutional terminology specific to the Bolivian health system.
(PDF)

S3 Appendix Interview guide for dog and cat owners, an instrument exploring pet owners’ views and experiences in southern Cercado. Colloquial and culturally embedded language is preserved to maintain contextual fidelity.
(PDF)

S1 Table Characteristics of health system personnel (n = 46) from decision-making entities interviewed in the qualitative component. This information was included to contextualize the community, although it was not analyzed in the main text.
(PDF)

S2 Table Sociodemographic characteristics of dog and cat owners; a table summarizing the gender, occupation, age, and years of residence of the 108 owners interviewed in the qualitative component. This information was included to contextualize the community, although it was not analyzed in the main text.
(PDF)
