# Supplementary material for: Fidelity of the implementation of antirabies vaccination for dogs and cats in the Plurinational State of Bolivia
Source: PLoS Negl Trop Dis. 2026 Jul 10;20(7):e0014535. doi: 10.1371/journal.pntd.0014535 (PMC13379090; doi:10.1371/journal.pntd.0014535)
Supplement: S1 Appendix — Language reflects local administrative and operational terms. (PDF) [file pntd.0014535.s001.pdf]

Material suplementario 1. Lista de chequeo para Entidades Tomadoras de Decisiones (ETDs)

	**Lista de chequeo**	
	**Determinar los factores que influyen en la fidelidad de la implementación de la vacunación antirrábica de perros y gatos en la zona sur del municipio de Cercado, Cochabamba (Bolivia).**	**Fecha:**<br>**Código:**

**Objetivo:** Evaluar la fidelidad de la implementación de la vacunación antirrábica de perros y gatos en centros de salud de la zona de estudio.				
	**Contenido**	**Si**	**No**	**Observaciones**
1	¿El número de puntos de vacunación antirrábica para perros y gatos fue distribuido según la población estimada?			
2	¿El número de brigadas dispuestas en la campaña de vacunación antirrábica para perros y gatos en la gestión 2021 fue el requerido según la población estimada?			
3	¿El número de canalizadores dispuestos en la campaña de vacunación antirrábica para perros y gatos fue el requerido según la población estimada?			
4	¿El número de vehículos con perifoneo dispuestos en la campaña de vacunación antirrábica para perros y gatos fue el requerido?			
5	¿El establecimiento de salud contó con la cantidad requerida de carnets de vacunación para perros y gatos?			
6	¿El establecimiento de salud contó con la cantidad requerida de cintillos de identificación para la vacunación de perros y gatos?			
7	¿El establecimiento de salud contó con la cantidad requerida de jeringas para la vacunación de perros y gatos?			
8	¿El establecimiento de salud contó con la cantidad requerida de planillas de registro para la vacunación de perros y gatos?			
9	¿El establecimiento de salud cuenta con cadena de frio para almacenar las vacunas antirrábicas para perros y gatos?			
10	¿El establecimiento de salud contó con la cantidad requerida de termos para la vacunación de perros y gatos?			
11	¿Los termos dispuestos para la campaña de vacunación antirrábica de perros y gatos son de tipo Kinselly?			
12	¿Se encuentran en buen estado los termos disponibles en el establecimiento de salud para la campaña de vacunación de perros y gatos?			

13	¿El personal de apoyo dispuesto para la campaña de vacunación antirrábica es capacitado previamente?			
14	¿El establecimiento de salud se encarga de la capacitación del personal de apoyo para la campaña de vacunación antirrábica?			
15	¿Las brigadas de vacunación se encuentran debidamente identificadas en el termo cuando salen a campo (ID y número de establecimiento)?			
16	¿Las brigadas de vacunación están conformadas por vacunador, registrado y canalizador?			
17	¿Los canalizadores que participaron en la ejecución de la campaña de vacunación antirrábica fueron dispuestos por las Fuerzas Armadas o Policía?			
18	¿Se realiza el marcado de domicilios con las señales de: V= Vacunado, R=Regresar o X=No hay mascotas?			
19	¿El personal dispuesto para conformar las brigadas de vacunación son estudiantes de medicina, enfermería o veterinaria?			
	**Cobertura**			
20	¿El establecimiento de salud contó con la cantidad requerida de dosis para la campaña de vacunación de perros y gatos?			
21	El establecimiento de salud alcanzo el 85% en la cobertura de vacunación antirrábica de perros y gatos el día dispuesto a la campaña masiva?			
22	¿El establecimiento de salud cuenta con la cantidad requerida de dosis para la vacunación diaria de perros y gatos por fuera de la campaña?			
	**Frecuencia**			
23	¿La difusión masiva de campaña en articulación con la estructura social en salud y los diferentes actores institucionales de la comunidad se realizó un mes antes de la campaña de vacunación para perros y gatos?			
24	¿Al año se realiza una campaña masiva de vacunación antirrábica para perros y gatos?			
25	¿Una vez finalizada la campaña masiva de vacunación antirrábica para perros y gatos, el establecimiento de salud realiza otras jornadas de vacunación?			
26	¿Es diaria la frecuencia con la que el establecimiento de salud realiza la vacunación antirrábica de perros y gatos?			
27	¿El establecimiento de salud realiza diariamente la vacunación antirrábica para perros y gatos?			
	**Duración**			
28	¿Cada jornada de vacunación tiene una duración de 8 horas?			

English Summary

Title: Checklist for Decision-Making Entities (ETDs).

Purpose: To assess factors influencing the fidelity of the anti-rabies vaccination campaign for dogs and cats in the southern zone of Cercado municipality, Cochabamba (Bolivia).

Description:

This checklist was used to evaluate the implementation of the rabies vaccination campaign across 17 health centers. It consists of 28 questions grouped into four components —content, coverage, frequency, and duration, based on national guidelines and relevant manuals. The checklist covers logistical, operational, and personnel-related aspects of the campaign (e.g., number of vaccination brigades, cold chain equipment, availability of identification materials, and daily vaccination practices). Responses are recorded as "YES" or "NO," with space for observations to identify barriers and facilitators. This tool helped determine the extent of adherence to planned activities and identified gaps that could impact vaccination coverage and campaign success.
