# Supplementary material for: Fidelity of the implementation of antirabies vaccination for dogs and cats in the Plurinational State of Bolivia
Source: PLoS Negl Trop Dis. 2026 Jul 10;20(7):e0014535. doi: 10.1371/journal.pntd.0014535 (PMC13379090; doi:10.1371/journal.pntd.0014535)
Supplement: S2 Appendix — Wording preserves institutional terminology specific to the Bolivian health system. (PDF) [file pntd.0014535.s002.pdf]

Material suplementario 2. Guía de entrevista semiestructurada para Entidades Tomadoras de Decisiones (ETDs)

	**Entrevista Semiestructurada**	
	**Determinar los factores que influyen en la fidelidad de la implementación de la vacunación antirrábica de perros y gatos en la zona sur del municipio de Cercado, Cochabamba (Bolivia).**	**Fecha:**
		**Código:**

**Objetivo:** Evaluar la fidelidad de la implementación de la vacunación antirrábica de perros y gatos en centros de salud de la zona de estudio.

Título del proyecto: Factores que influyen en la fidelidad de la implementación de la vacunación antirrábica de perros y gatos en la zona sur del municipio de Cercado, Cochabamba (Bolivia).

Tipo de entrevista

Entrevista semiestructurada.

Tiempo estimado

Entre 1,5 y 2 horas.

Objetivo de la entrevista: Conocer las percepciones, creencias, conocimientos y opiniones que los diferentes integrantes de las instituciones tomadoras de decisiones tienen sobre aspectos relacionados con la vacunación antirrábica para perros y gatos en la zona sur del municipio de Cercado.

Pregunta de investigación: ¿Cuáles son los factores que influyen en la fidelidad de la implementación de la vacunación antirrábica de perros y gatos en la zona sur del municipio de Cercado, Cochabamba (Bolivia)?

Categorías: Conocimientos sobre la Rabia humana y Rabia animal, conocimientos sobre la planificación y ejecución de la intervención, factores del profesional de salud, factores de la norma nacional de profilaxis para Rabia humana y animales domésticos, el manual para campañas de vacunación contra la Rabia canina y felina, y factores administrativos.

Instrumento: Guía de entrevista semiestructurada.

Buenos días. Mi nombre es Andrés Rodrigo Gómez Vidal, actualmente soy estudiante de la Universidad de Antioquia en Colombia. Para comenzar, quiero agradecerle por brindarme este tiempo para poder realizar esta entrevista. Además de ello, quisiera saber si podría permitirme grabar la sesión para realizar la trascripción y, posteriormente, el análisis de la información que usted me brinde. Toda la información se mantendrá de forma anónima.

Actualmente me encuentro trabajando con el tema de la Rabia en perros y gatos, puntualmente en lo relacionado con la planificación y ejecución de la campaña de vacunación antirrábica en la zona sur del municipio de Cercado. Usted, al trabajar desde la parte administrativa o asistencial, tiene una experiencia y conocimientos que son de interés para esta investigación.

Preguntas:

Percepciones acerca del sistema de atención en salud

1. ¿Cómo ha sido su comunicación con las diferentes entidades tomadoras de decisiones al momento de realizar la planificación de la campaña de vacunación antirrábica?

2. ¿Cómo ha sido su comunicación con las organizaciones sociales y juntas vecinales de la zona sur del municipio?

3. ¿Ha tenido dificultades para acceder a los diferentes barrios y OTBs que representan a la organización de juntas vecinales? En caso de respuesta afirmativa, ¿de qué tipo han sido esas dificultades?

4. ¿Ha recibido información clara y oportuna sobre las acciones y decisiones que los vecinos consideran importantes al momento de planificar la campaña de vacunación antirrábica para perros y gatos?

Redes de intercambio de información:

5. ¿Con cuáles personas de las organizaciones sociales puede hablar sobre temas relacionados con la rabia y su medida preventiva?

6. ¿En qué lugares la población puede obtener información sobre la Rabia y las medidas de prevención que debe considerar?, ¿esta información se brinda solamente en español o se la brinda también en idioma nativo como el quechua?

Conocimientos, comportamientos y actitudes frente a la Rabia:

7. Cuando escucha hablar sobre Rabia, ¿qué es lo primero que piensa?

8. Cuando escucha hablar sobre mordeduras de perros o gatos, ¿qué es lo primero que piensa?

9. ¿Qué sabe usted sobre la Rabia?

10. ¿Qué haría usted si se entera que alguien de su familia o entorno personal ha sido agredido por un perro o gato?

11. ¿Qué piensa usted de una persona que exige el certificado de vacunación a los propietarios de perros y gatos vecinos a su domicilio?

Roles productivos:

12. ¿Qué labores y actividades son propias de la institución donde se desempeña laboralmente en relación con el control y vigilancia de la Rabia?

13. ¿Considera que las acciones que realizan como entidad tomadora de decisiones son suficientes para poder controlar la circulación del virus de la Rabia?

14. ¿Considera importante tener otros mecanismos de control para la población en general, considerando la importancia de la Rabia para la salud?

15. Según usted ¿por qué existe una diferencia entre la zona sur y la norte del municipio, considerando la constante aparición de casos de Rabia en perros o gatos?

English Summary

Title: Semi-structured Interview Guide for Decision-Making Entities (ETDs).

Purpose: To identify factors influencing the fidelity of the rabies vaccination campaign for dogs and cats in the southern zone of Cercado municipality, Cochabamba (Bolivia).

Description:

This guide was used for semi-structured interviews with decision-makers involved in the rabies vaccination campaign. The interview lasts approximately 1.5 to 2 hours and explores perceptions, knowledge, behaviors, and attitudes related to the planning and execution of the campaign. Questions cover topics such as communication with community and health entities, information sharing networks, perceptions and knowledge of rabies, and institutional roles and responsibilities. These interviews aim to capture barriers and facilitators in implementing vaccination strategies and highlight differences between the southern and northern zones of the municipality.
