# Supplementary material for: Fidelity of the implementation of antirabies vaccination for dogs and cats in the Plurinational State of Bolivia
Source: PLoS Negl Trop Dis. 2026 Jul 10;20(7):e0014535. doi: 10.1371/journal.pntd.0014535 (PMC13379090; doi:10.1371/journal.pntd.0014535)
Supplement: S3 Appendix — Colloquial and culturally embedded language is preserved to maintain contextual fidelity. (PDF) [file pntd.0014535.s003.pdf]

Material suplementario 3. Guía de entrevista semiestructurada para tutores de perros y gatos en la zona sur del municipio de Cercado, Bolivia.

<table>
<tr><td rowspan="4"></td><td colspan="3">Entrevista a la comunidad</td><td>Fecha:</td></tr>
<tr><td colspan="3">Evaluar la fidelidad de la implementación de la vacunación antirrábica de perros y gatos en la zona sur del municipio de Cercado, Cochabamba (Bolivia).</td><td></td></tr>
<tr><td>Sexo</td><td>Edad</td><td>Profesión</td><td>Tiempo de vivir en la zona</td></tr>
<tr><td></td><td></td><td></td><td></td></tr>
<tr><td colspan="5">GUIÓN DE ENTREVISTA</td></tr>
<tr><td colspan="5">Estimado señor/señora. Estamos llevando a cabo este estudio como parte de una investigación. Buscamos recopilar información sobre el programa de “Vacunación antirrábica para perros y gatos” a través de la opinión de la comunidad. Con su permiso le haremos algunas preguntas, tomando un máximo de 30 minutos. ¿Acepta participar? Sí/--/ No /--/

• ¿Considera la Rabia un problema grave? ¿Por qué?
• Si usted o sus familiares fueran mordidos por un perro o gato, ¿qué medidas toman?
• ¿De quién cree que es la responsabilidad de realizar la campaña de vacunación antirrábica de perros y gatos?
• ¿Usted como ciudadano como puede contribuir a prevenir la Rabia?
• ¿Piensa que la vacunación antirrábica para perros y gatos es útil en la prevención y control de la Rabia?
• ¿Tuvo algún percance en la última campaña de vacunación antirrábica realizada en la zona?
• ¿Qué sugerencias podría dar para mejorar la vacunación antirrábica para perros y gatos?
• Durante el proceso de vacunación: ¿sus preguntas fueron contestadas apropiadamente?
• ¿Qué los motivaría a realizar la vacunación antirrábica de sus perros y gatos?
• ¿Participaría, junto con los centros de salud, en la vacunación antirrábica para perros y gatos?

Con esto daríamos fin a la entrevista y agradezco su participación.</td></tr>
</table>

English Summary

Title: Semi-structured Interview Guide for Dog and Cat Owners in the Southern Zone of Cercado Municipality, Bolivia.

Purpose: To evaluate the fidelity of the rabies vaccination campaign for dogs and cats through the perspective of community members in southern Cercado.

Description:

This semi-structured interview guide, lasting about 30 minutes, was designed to gather insights from adult dog and cat owners on their perceptions of rabies as a public health issue and their experiences with the vaccination campaign. Questions explore perceptions of rabies risk, responsibility for vaccination campaigns, community contributions to rabies prevention, and experiences during past campaigns (e.g., challenges, suggestions for improvement, motivation to vaccinate pets). The instrument also investigates trust in health centers and readiness to participate in future campaigns alongside local health entities.
