# Supplementary material for: Fidelity of the implementation of antirabies vaccination for dogs and cats in the Plurinational State of Bolivia
Source: PLoS Negl Trop Dis. 2026 Jul 10;20(7):e0014535. doi: 10.1371/journal.pntd.0014535 (PMC13379090; doi:10.1371/journal.pntd.0014535)
Supplement: S1 Table — This information was included to contextualize the community, although it was not analyzed in the main text. (PDF) [file pntd.0014535.s004.pdf]

S1 Table. Characteristics of health system personnel from decision-making entities in the Cercado municipality, Cochabamba Province (Plurinational State of Bolivia) (n=46), 2021.

Variables	Frequency (percent)
Gender	
Male	18 (39.1%)
Female	28 (60.9%)
Occupation	
Medical doctor	18 (39.1%)
Veterinarian	9 (19.5%)
Nurse	17(37%)
Social communicator	1 (2.2%)
Operational technician	1 (2.2%)
