# Supplementary material for: Fidelity of the implementation of antirabies vaccination for dogs and cats in the Plurinational State of Bolivia
Source: PLoS Negl Trop Dis. 2026 Jul 10;20(7):e0014535. doi: 10.1371/journal.pntd.0014535 (PMC13379090; doi:10.1371/journal.pntd.0014535)
Supplement: S2 Table — This information was included to contextualize the community, although it was not analyzed in the main text. (PDF) [file pntd.0014535.s005.pdf]

S2 Table. Characteristics of dog and cat owners and residents in the southern area of the Cercado municipality, Cochabamba Province (Plurinational State of Bolivia) (n=108), 2021.

**Variables**	**Frequency (percent)**
Gender	
Male	51 (47.2%)
Female	57 (52.8%)
Occupation	
Student	49 (45.4%)
Trader/Business Owner	16 (14.8%)
Professional	43 (39.8%)
Age (in years)	
18-26	50 (46.3%)
27-59	51 (47.2%)
>60	7 (6.5%)
Time living in the area (in years)	
1-10	56 (51.8%)
11-20	33 (30.5%)
21-30	14 (13.0%)
31-40	4 (3.7%)
>40	1 (1.0%)
